# Evaluating the Effectiveness of a School-Based Cognitive Behavioural Therapy Intervention for Anxiety in Adolescents Diagnosed with Autism Spectrum Disorder

**DOI:** 10.1007/s10803-016-2857-7

**Published:** 2016-07-20

**Authors:** Sarah Luxford, Julie A. Hadwin, Hanna Kovshoff

**Affiliations:** 1 Developmental Brain-Behaviour Laboratory, Department of Psychology, University of Southampton, Highfield, Southampton, S017 1BJ UK; 2 Present Address: Oxfordshire Educational Psychology Service, Samuelson House, Tramway Road, Banbury, Oxford, OX16 5AU UK

**Keywords:** Autism, Anxiety, CBT, Social worry, Attentional control, Attention to threat

## Abstract

This study evaluated the effectiveness of a school-based Cognitive Behavioural Therapy (CBT) on symptoms of anxiety, social worry and social responsiveness, and indices of attentional control and attentional biases to threat in adolescents diagnosed with Autism Spectrum Disorder. Thirty-five young people (11–14 years; IQ > 70) with ASD and elevated teacher or parent reported anxiety were randomly assigned to 6 sessions of the Exploring Feelings CBT intervention (Attwood in Exploring feelings (anxiety). Future Horizons, Arlington, 2004) (n = 18) or a wait-list control group (n = 17). The intervention (compared to the wait-list control) group showed positive change for parent, teacher and self-reported anxiety symptoms, and more marginal effects of increased teacher-reported social responsiveness. The discussion highlights the potential value and limitations of school-based CBT for young people with ASD.

## Introduction

Progression in our understanding of Autism Spectrum Disorder (ASD) has led to its consideration as a range of abilities and difficulties that affect social communication and repetitive and restrictive behaviours (American Psychiatric Association 2013). In addition to the impairments typically associated with ASD, several studies have estimated that around half of children and adolescents also meet the diagnostic criteria for an anxiety disorder (reviews by Kerns and Kendall 2012; Simonoff et al. 2008). Moreover, reported prevalence rates are considerably higher than in typically developing children (Kerns and Kendall 2012) and in children with specific learning disabilities (Gillott et al. 2001). In school settings teachers have also reported that anxiety-related issues are among the most common presenting problems for young people with ASD (Waddington and Reed 2006). Further research has found that these difficulties impact on social functioning and academic performance (Bellini 2004; Reaven et al. 2009; Sze and Wood 2007). For example, Sukhodolsky et al. (2008) found significant positive associations between negative social experiences in school and anxious affect in children with ASD. Researchers have suggested that the relationship between anxiety and social difficulties in school is bi-directional; the presence of anxiety contributes to, as well as results from, the challenges experienced by many children and adolescents with ASD (review by White et al. 2013).

Given the prevalence and impact of anxiety in young people with ASD, treatment approaches for this population have received increased empirical attention. One treatment option for young people with ASD is cognitive-behavioural therapy (CBT; Beck et al. 1979). A fundamental principle of CBT is to address the behavioural manifestations of anxiety, as well as the underlying negative cognition often associated with anxious affect (Rotheram-Fuller and MacMullen 2011). It is proposed to provide individuals with an opportunity to learn skills to challenge dysfunctional beliefs and replace them with more adaptive and positive thinking (Beck 1993). CBT continues to be a primary treatment recommendation for anxiety disorders (National Institute for Health and Clinical Excellence, NICE 2013). Several recent reviews have highlighted its efficacy (compared to wait-list controls) for the treatment of anxiety in typically developing children and adolescents (e.g., James et al. 2013) and those diagnosed with ASD (Kreslins et al. 2015; Sukhodolsky et al. 2013).

In addition to assessing the effect of CBT on anxiety symptoms, further research has explored its broader impact on attention and behaviour. Several frameworks suggest that increased anxiety is associated with poor attentional control and selective attention or hypervigilance for the detection of environmental threat (review by Richards et al. 2014). Recent studies have found that CBT can have a positive impact on the reduction of threat biases and poor attentional control typically associated with anxious affect (Hadwin and Richards 2016; Malowsky et al. 2010; Reinholdt-Dunne et al. 2015). Consistently, further findings show that asking individuals to suppress negative reactions to aversive stimuli led to increased activity in brain regions associated with attentional control and less activation in those linked to negative affect (Ochsner et al. 2002). Further evidence indicates that CBT can lead to reduced anxiety and fewer negative thoughts in typically developing young people (Waters et al. 2008) and those diagnosed with ASD (Chalfant et al. 2007; Sofronoff et al. 2005). With respect to changes in behaviour more broadly, Storch et al. (2013) reported reductions in anxiety following CBT, in addition to improved parent-reported social functioning.

While emerging findings have been encouraging, there are still relatively few studies that have assessed the effectiveness of CBT for young people with ASD and these have typically been explored in clinic-based settings. Given that children and adolescents with ASD can show difficulty in generalising learned skills to new contexts (Bellini et al. 2007), it is important to consider whether schools might be an effective context for the delivery of CBT interventions. The use of school-based CBT for anxiety in typically developing children is well supported (for a review see Neil and Christensen 2009) and researchers have suggested methods for adapting school-based interventions for use with pupils with ASD (Rotheram-Fuller and MacMullen 2011). This agenda has become increasingly significant in the context of an increased focus in the UK over the last two decades towards inclusive education, where schools are expected to educate all pupils within a mainstream setting and to “actively seek to remove the barriers to learning and participation that can hinder or exclude pupils with special educational needs (Department for Education 2001, p. 5).” While this initiative allows all students to receive support to meet their potential in a conventional school environment, research has shown that pupils with ASD can find inclusion anxiety-provoking, particularly at secondary level (Browning et al. 2009; Humphrey and Lewis 2008), highlighting the need to develop initiatives within schools to support inclusion.

The current study used a randomised control trial (RCT) to measure the effectiveness of a school-based CBT intervention (versus a wait-list control) on the reduction of anxiety symptoms (including social worries) in adolescents diagnosed with ASD. In addition, it aimed to provide preliminary evidence to explore the broader impact of a CBT intervention on social responsiveness, as well as attentional control and attention to threat. It was anticipated that pupils in the intervention group would experience a significantly greater reduction in anxiety in comparison to the wait-list control group, who attended mainstream school as usual. We used multiple informants (teachers, parents and self-reported) to provide an accurate and robust picture for anxiety change (see Kasari et al. 2012). In addition, we anticipated that the intervention would have broader benefits with respect to increased social responsiveness and increased attentional control (i.e., lowered levels of distraction and reduced attention to threat).

## Method

### Participants

**Fig. 1:**
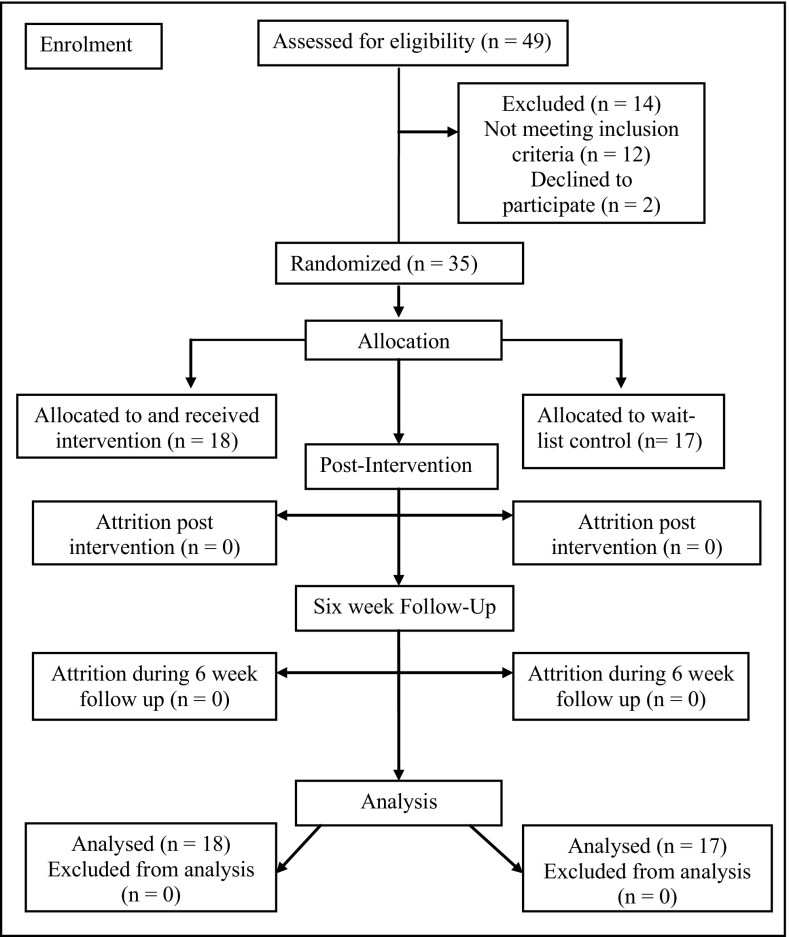
Flow of participants through each stage of the study

The participants included 35 pupils from four mainstream secondary schools located in the south-east of England (*Mean age* = 13.2, *SD* = 1.1, range 11.10–15.80; 31 boys). Participants were required to have a formal diagnosis of ASD from a qualified health professional (*N* = 26 adolescents had a formal diagnosis of ASD and *N* = 9 had a diagnosis of Asperger’s Syndrome). To address variance in the time since diagnosis (range 6 months to 13 years), the Social Communication Questionnaire (SCQ; Rutter et al. 2003a, b) was used to confirm that pupils met the criteria for ASD. Participants were also required to have a verbal and total IQ score of ≥70 and to be currently experiencing clinically significant symptoms of anxiety, as measured by elevated scores for either teacher reported school anxiety (score > 17; Lyneham et al. 2008) or parent reported anxiety (score > 24 on the Spence Children’s Anxiety Scale; Spence 1998). These scores were used as baseline measures of anxiety. Pupils who were identified as being in active treatment or currently receiving medication for anxiety (n = 3) were excluded from the study. To be included all pupils met the requirement of attending a minimum of 5 of the 6 intervention sessions. Figure 1 outlines the flow of participants through the study.

### Measures

#### Social Communication Questionnaire (Rutter et al. 2003a, b)

The social communication questionnaire is a 40-item parent-report measure used to assess and screen for characteristics of ASD in the previous 3 months. It is designed for use with participants aged 4–40 years and each item requires a yes–no response (score range 0–39). It has established validity with the Autism Diagnostic Interview-Revised (ADI-R; Rutter et al. 2003a, b) and has been shown to discriminate reliably between children with and without ASD at the established cut-off point of (≥15; see Berument et al. 1999), with a sensitivity of 0.88 and a specificity of 0.72 (Chandler et al. 2007).

#### Wechsler Abbreviated Scale of Intelligence: Second Edition (WASI; Wechsler 1999)

The measure was designed for individuals aged 6 to 89 years and consists of four subtests that are totalled to create a score for performance, verbal and full scale intelligence. The WASI has good internal reliability (0.98) and test–re-test reliability (0.92; Garland 2005).

### Primary Outcome: Anxiety Measures

#### School Anxiety Scale (Lyneham et al. 2008)

The school anxiety scale is a 16-item teacher-reported measure of anxiety designed to assess the behaviour of children at school from 5 to 12 years of age. Items are answered on a four-point scale. The measure provides a total score for anxiety (scores ranging from 0–48). It includes two subscale scores (reflecting social anxiety and generalised anxiety), and in the current study we used the total anxiety score (in the current sample the reliability was good, *α* > 0.7).

#### Spence Anxiety Scale (Spence 1998)

We used self-reported and parent-reported versions of the Spence anxiety scale to measure adolescent anxiety symptoms. The questionnaire was designed for use with 7–16-year-olds and includes 38 items that assess anxiety symptoms based on DSM-IV anxiety disorder subtypes (American Psychiatric Association 1994). It also includes six positive filler items to reduce negative response bias. For each item, children and parents are asked to rate child symptoms based on the descriptions given on a four-point Likert scale (score range 0–114). The questionnaire has high internal consistency and satisfactory test–retest reliability (in the current study *α*s > 0.7 for self and parent reported).

#### Social Worries Questionnaire (Spence 1995)

The social worries questionnaire includes self- and teacher-reported versions and was developed to assess symptoms of social anxiety. It contains 13 items relating to worry about and avoidance of social-evaluative situations that are rated in terms of worry experienced in each situation (score = 0–26). The measure is reported to have high internal consistency (Russell and Sofronoff 2005 and α > 0.7 in the current study).

### Secondary Outcome Measures

#### Social Responsiveness Scale (Constantino and Gruber 2002)

The social responsiveness scale is a 65 item rating scale developed for children and adolescents aged 4–18 years and measuring behaviours associated with social impairment based on parent and teacher reported. Items are scored from 1 (not true) to 4 (almost always true) and the total score = 0–260, where higher scores reflect greater severity of social difficulty. Internal consistency and stability are both excellent (see Constantino et al. 2003).

#### Attentional Control

We measured attentional control using a variation of the Erikson flanker task (Eriksen and Schultz 1979). This measure assesses an individual’s ability to focus attention to identify whether a centrally presented arrow is pointing left or right and ignore flanker arrow heads. Flankers are either consistent with (congruent condition) the direction of the central arrow, pointing in the opposite direction (incongruent condition) or have no relationship to the central stimuli (neutral condition; see Rueda et al. 2004). In the current task, each display appeared immediately after a (500 ms) fixation cross, and remained on screen until either the participant made a response or 1500 ms passed. All participants completed 12 practice trials before performing 3 blocks of test trials, each consisting of 48 individual trials and with 16 trials of each type (congruent, incongruent and neutral) presented in a random order. The overall task took around 10 min for each child. No feedback was provided for correct or incorrect answers. On each trial, accuracy and response time was recorded. Preliminary analyses looked at reaction times (RTs) for each trial type; however, the focus of the analysis for this task was a conflict score, calculated by subtracting the mean RT of the congruent items from the mean RT of the incongruent items. Higher scores are indicative of greater distractor interference or distractibility (Rueda et al. (2004).

#### Attention to Threat

In order to explore attention to threat, an emotional stroop colour matching schematic face task was used (see Hadwin et al. 2009). Angry, happy, fear and neutral face schematic face stimuli were employed, with each face being made up of a pair of eyes, eyebrows and a mouth. The face outline was red, blue, green or yellow. The presentation screen was black. Participants saw 24 trials for each emotion; 12 emotion faces and 12 inverted face control trials, making a total of 72 randomly presented trials. Inverted faces were used to ensure that responding reflects interference of emotional stimuli and not face parts. Upright and inverted face stimuli were presented individually and in the same position on the screen, remaining on screen until either the participant made a response or 1500 ms passed. Participants were asked to match the outline colour of a picture on the screen to the coloured buttons as quickly and accurately as possible. The responses were made using a response box that included red, blue, green and yellow buttons positioned in a fixed order from left to right. On each trial, accuracy and response time was recorded. To address task validity, preliminary analyses looked at reaction times (RTs) for each trial type. Attentional bias scores were calculated by subtracting individual mean RT values for colour matching in neutral faces from matching angry, happy and fear faces. Positive scores indicate increased interference to colour matching for emotion versus neutral faces and negative scores facilitation and bias scores that tend to zero indicate that there was no difference between colour matching between faces.

### CBT Intervention

We used the ‘Exploring Feelings’ manualized CBT intervention created by Attwood (2004). This 6-week programme uses developmentally appropriate language and materials designed for use with pupils with ASD. Each of the six sessions lasted for 90 min and in the current study was led by the same researcher in all four settings. The intervention delivery was supported by a teaching assistant (TA) in each school, who participated in the sessions and retained regular contact with the pupils outside of the sessions. This approach enabled the TA to reinforce strategies learned within CBT sessions across the school day, and to remind and encourage the pupils to use learned strategies when they were experiencing elevated anxiety. This delivery model is unique in its active targeting of generalisation skills outside of the CBT session, in a naturalistic environment.

At the end of each session, a home project was explained to participants and discussed at the start of the next session. Worksheets for the sessions were taken home on completion of the intervention. The CBT programme is designed to be highly structured and informative and the participants work to create a metaphoric ‘tool box’ of anxiety management strategies across sessions.

### Procedure

Ethical approval was obtained from the University ethics’ committee and research governance. In the first stage of recruitment, the researcher approached all secondary schools located within one district in the south of England (N = 9) and provided information regarding the study. Four schools indicated interest in participating, and were asked to identify all pupils attending the schools with an ASD diagnosis. Forty-nine adolescents with ASD were identified by school personnel and the study information was sent home for parents to consider. Following informed written consent from 47 parents, they completed the questionnaires related to social communication, social responsiveness and anxiety. Educational records were checked to confirm formal ASD diagnosis. Parents of adolescents who did not meet the inclusion criteria to take part in the study were informed directly by the researcher (n = 12).

Form tutors who spend extended time with the pupils each day and receive feedback from staff regarding the pupils’ performance and well-being were asked to complete the social responsiveness scale, school anxiety and social worries questionnaires. The same teacher completed these measures at all three time points. The researcher then met individually with pupils to administer the WASI, the attention tasks, and the anxiety and social worries questionnaires. Informed assent was received from all adolescent participants prior to completing the pre-measures.

Following completion of the pre-intervention measures, a total of 35 participants were available for the study. Participants within each school were randomly assigned through a computer-generated assignment system to either the intervention group (*N* = 18) or the wait-list control group (*N* = 17). Intervention groups contained between four and six participants (see Sofronoff et al. 2005). Four groups participated in the study over a 3-month period–consisting of two groups running simultaneously for 6 weeks, and a 6-week follow up. Participants assigned to the wait-list control group were given the opportunity to receive the intervention as delivered by schools after the study was completed. Administration of measures for participants in school took between 40 and 60 min at each time point and the order of experimental tasks was randomised.

## Results

### Approach to Analysis

To explore the impact of the ‘Exploring Feelings’ CBT intervention on the primary (anxiety and social worry) and secondary outcome measures (social responsiveness, attentional control and attention to threat), group differences were explored over three time points using a repeated measures ANOVA with 2 Group (Intervention and Wait-list control) and 3 Time: pre-intervention (T1), post-intervention (T2) and follow-up (T3). Raw scores from questionnaire data were analysed for anxiety and social responsiveness, conflict scores were computed representing attentional control and bias scores for attention to threat. Effect sizes are reported for all analyses and we only report significant post hoc analyses (and after adjusting the *p* value to address multiple comparisons).

### Descriptive Statistics

The respective means for the full scale IQ for the intervention and wait-list control groups were 105.44 (*SD* = 17.83, range 76–157) and 102 (*SD* = 11.30, range 82–124). The mean SCQ scores for the intervention and control groups were 18.61 (*SD* = 4.33, range 15–28) and 19.06 (*SD* = 4.94, range 15–30). There was no mean group difference for IQ or SCQ scores (in both cases *t* < 1 and *p* > 0.1). There was no attrition for pupil and teacher responses across the three time points. Parent-responses were obtained for all participants at pre- and post-intervention (T1). At follow-up (T3), responses were not received from 3 parents of participants in the intervention group and 7 in the control group (analyses are, therefore, reported with reduced participant numbers).

Means, standard deviations and range of scores for the primary and secondary variables at T1, T2 and T3 are shown in Table 1. There were no significant group differences between any scores on self-reported anxiety, social worry, social responsiveness, attentional control and attention to threat (all *ts* < 1.5 and all *ps* > 0.1). There were, however, significant differences for parent-reported anxiety *t*(33) = 2.47, *p* = 0.01 and teacher-reported school anxiety *t*(33) = 2.88, *p* < 0.01, with increased baseline scores for the intervention group. To address these T1 differences all analyses were repeated with T1 entered as a covariate.

**Table 1:** Mean (standard deviation) [range] for self, parent teacher reported measures at each time point

	Intervention group (n = 18)^#^			Wait-list control group (n = 17)		
Variable	T1	T2	T3	T1	T2	T3
*Anxiety symptoms*						
Parent	47.61 (16.25) [16–78]	31.89 (14.86) [10–62]	26.67 (10.68) [13–51]	35.5 (10.82) [15–50]	40.94 (16.03) [18–74]	40.82 (19.05) [3–73)
Self	40.50 (16.87) [15–87]	27.50 (14.70) [10–57]	26.82 (15.50) [4–49]	35.12 (15.32) [10–77]	35.41 (21.35) [15–100]	30.35 (14.62) [5–66]
Teacher	28.61 (7.81) [9–39]	18.94 (8.93) [3–38]	14.39 (7.74) [2–34]	20.29 (9.23) [7–48]	20.82 (9.81) [10–48]	19.94 (11.23) [5–48]
*Social worry*						
Self	12.33 (4.74) [4–22]	8.83 (4.42) [3–16]	7.35 (4.82) [0–15]	12.41 (5.75) [4–26]	12.29 (6.62) [4–24]	9.76 (6.80) [1–24]
Teacher	11.28 (3.611) [5–16]	8.00 (4.42) [0–16]	6.39 (3.13) [0–14]	9.18 (4.28) [0–15]	8.41 (4.45) [1–15]	8.41 (5.01) [0–16]
*Social responsiveness*						
Parent	111.83 (25.24) [37–152]	98.56 (23.67) [53–138]	96.47 (21.69) [66–132]	114.06 (23.72) [69–51]	109.41 (24.68) [69–50]	103.08 (13.81) [84–26]
Teacher	96.56 (31.44) [35–152]	87.94 (29.12) [27–159]	83.11 (35.40) [18–163]	89.24 (37.79) [27–159]	92.88 (37.80) [29–159]	92.29 (35.00) [14–159]
*Attentional control scores*						
	194.81 (108.81) [420]	67.06 (38.62) [122]	48.10 (45.79) [169].	206.06 (137.08) [524]	151.18 (149.86) [451]	134.56 (93.76) [292]
*Emotional stroop bias scores*						
Happy	−27.58 (99.84) [363]	−0.29 (66.08) [295]	−19.26 (54.62) [225]	8.35 (121.01) [526]	−7.06 (77.53) [295]	−2.70 (100.98) [466]
Fear	−2.55 (155.40) [679]	21.21 (87.47) [352]	−9.64 (56.08) [210]	30.46 (97.64) [437]	−11.19 (79.76) [296]	19.25 (80.82) [287]
Angry	97.25 (127.88) [419]	13.25 (62.65) [236]	−9.24 (42.47) [141]	64.01 (69.18) [320]	23.06 (105.90) [493]	44.82 (163.19) [581]

For parent reported measures at T3 N = 15 for the intervention group and N = 11 for the control group

Correlations between all primary and secondary T1 measures are shown in Table 2.^1^ This table indicates that parent- and self-reported anxiety measures were significantly correlated. Significant positive correlations were also found between self-reported anxiety and social worry. Parent and teacher-reported anxiety measures also significantly correlated with their own reports of social responsiveness, and parent reported social responsiveness was associated with higher child IQ. The flanker conflict score did not correlate with any of the primary outcomes, though increased IQ was associated with greater attentional control (i.e., less interference) in this task. For the threat appraisal task, parent-reported anxiety was correlated with angry bias scores, indicating that as anxiety increases, response times to colour match angry versus neutral faces increased (the interference of threat stimuli on colour matching with increased anxiety and this result was not evident for happy and fearful faces; see Table 2; Fig. 3).

**Table 2:** Summary of correlations at Time 1 between parent, pupil and teacher-reported measures

	1	2	3	4	5	6	7	8	9	10	11	12
*Anxiety*												
1. Parent	–	0.49**	0.24	0.32	0.11	0.39*	0.14	−0.22	−0.14	0.44**	−0.19	0.27
2 Self		–	0.14	0.48**	0.11	0.30	0.25	0.20	−0.09	0.26	−0.08	−0.26
3 Teacher			–	0.17	0.73**	−0.07	0.45**	−0.13	−0.13	−0.08	−0.20	−0.12
*Social worry*												
4 Self				–	0.12	0.25	0.24	−0.06	0.04	0.18	0.21	0.08
5 Teacher					–	0.19	0.39*	−0.08	−0.18	−0.10	−0.09	−0.13
*Social responsiveness*												
6 Parent						–	0.12	−0.28	−0.08	0.32	0.01	0.37*
7 Teacher							–	−0.21	0.03	−0.21	−0.17	0.04
*Attentional control*												
8. AC								–	−0.15	0.12	0.05	−0.40*
*Emotional stroop bias*												
9 Happy									–	0.14	0.69**	0.12
10 Angry										–	0.25	0.38*
11 Fear											–	0.13
12 IQ												–

* *p* < 0.05; ** *p* < 0.001

### Primary Outcomes

#### Parent-Reported Anxiety

**Fig. 2:**
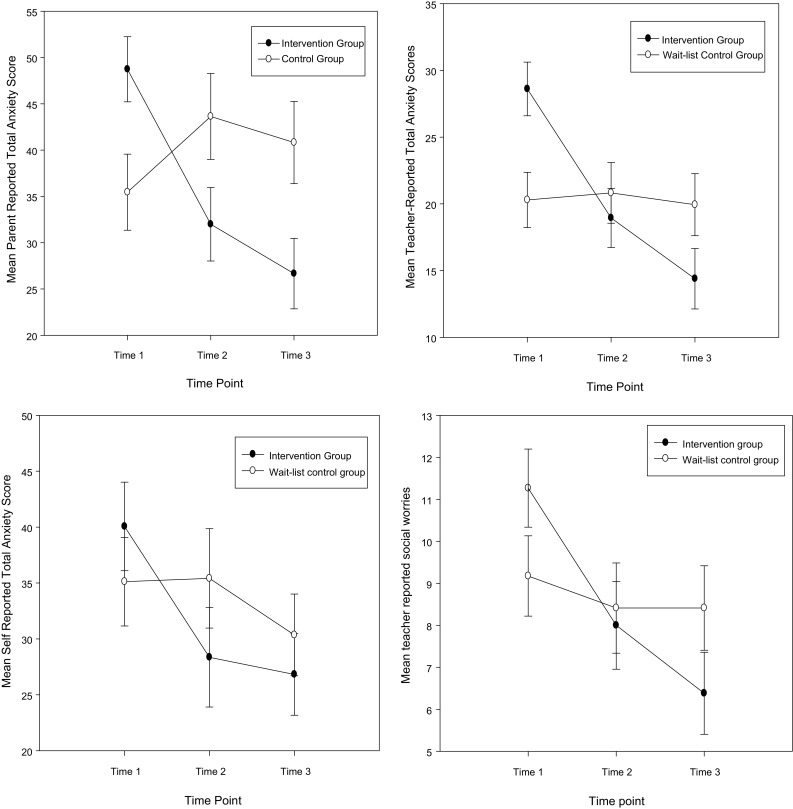
Mean parent and self-reported anxiety symptoms, teacher-reported school anxiety and social worry symptoms at each time point for the intervention and wait-list control group

Considering change across all three time points (and with a reduced sample size at T3 for the intervention (n = 15) and control group (n = 11), the analysis showed a main effect of time [*F*(2, 24) = 5.08, *p* = 0.01, *η*_*p*_^2^ = 0.18], highlighting marginal differences between T1 (*M* = 43.12, *SD* = 14.94) with T2 (*M* = 36.93, *SD* = 16.16) and significant T1–T3 (*M* = 32.65, *SD* = 16.12) differences (T2–T3 ns). The main effect of group was not significant (*F* < 1 and *p* > 0.1). There was also an interaction between time and group [*F*(2, 24) = 16.74, *p* < 0.001, *η*_*p*_^2^ = 0.41]. Post-hoc analyses indicated that within groups parent-reported anxiety symptoms were significantly different for the intervention group between each time point. There were no within group differences for the control group. Considering group differences at each time point, these were significantly different at T1 (intervention group > control group) and T3 (intervention group < control group; see Fig. 2).^2^

#### Self-Reported Anxiety

This analysis showed a significant main effect for Time (*F* (2,64) = 9.71, *p* < 0.001,*η*_*p*_^2^ = 0.23), highlighting differences in anxiety symptoms between T1 (*M* = 37.59, *SD* = 16.27) with T2 (*M* = 31.88, *SD* = 18.40 and T1 with T3 (*M* = 28.59, *SD* = 14.95; T2–T3 ns). There was no significant group effect (*F* < 2, *p* > 0.5). A significant interaction between group and time was also found (*F*(2,64) = 4.45, *p* = 0.015, *η*_*p*_^2^ = 0.12); indicating a significant reduction in anxiety from T1–T2 and T1–T3 for the intervention group (T2–T3 ns) and no significant changes were found for the control group over time (see Fig. 2).

#### Teacher-Reported School Anxiety

This analysis showed a main effect of time [*F*(2,33) = 10.27, *p* < 0.01, *η*_*p*_^2^ = 0.24], indicating significant differences in teacher reported school anxiety between T1 (*M* = 10.26 *SD* = 4.03) with T2 (*M* = 8.20, *SD* = 4.37) and T3 (*M* = 7.37, *SD* = 4.22) (T2–T3 ns). There was also a significant interaction between group and time (*F* (2,33) = 5.23, *p* < 0.01, *η*_*p*_^2^ = 0.14). Post-hoc analyses showed that for the control group there were no differences between any time points. In contrast, the intervention group showed differences between T1 scores compared with T2 and T3 scores (T2–T3 ns; see Fig. 2). The main effect of group was not significant (*F* < 1 and *p* > 0.1).^3^

#### Social Worry

A repeated measures ANOVA was conducted on the Social Worries Questionnaire (self-reported and teacher versions). For the pupil version, a significant main effect for time was found [*F*(2,33) = 10.43, *p* < 0.001, *η*_*p*_^2^ = 0.25] indicating significant improvements in anxiety symptoms from T1 (*M* = 12.37, *SD* = 5.17) to T2 (*M* = 10.51, *SD* = 5.78) and T3 (*M* = 8.56, *SD* = 5.93) and between T2 and T3. (Note that the T1–T2 difference became non-significant when correcting for multiple comparisons). There was no main effect of group and no significant interaction between group and time (in both cases *F* < 2 and *p* > 0.1). For the teacher version, a significant main effect of time was also found [*F*(2,32) = 10.27, *p* < 0.001, *η*_*p*_^2^ = 0.24], showing improvements from T1 (*M* = 10.26, *SD* = 4.03) to T2 (*M* = 8.20, *SD* = 4.37) and T3 (*M* = 7.37, *SD* = 4.21) (T2–T3 ns). There was no significant group effect (*F* < 2, *p* > 0.5). There was a significant interaction between time and group (*F* (2,32) = 5.23, *p* < 0.01, *η*_*p*_^2^ = 0.14), highlighting a significant reduction in teacher reported social worry between all time points for the intervention group. No significant differences were found for the control group between any time points (see Fig. 2).

### Secondary Outcomes

#### Social Responsiveness

For parent reported and considering analysis across all three time points, there was a main effect of time (*F* (2, 25) = 15.69, *p* < 0.001, *η*_*p*_^2^ = 0.37) highlighting increased social responsiveness (as reflected in lowered scores) at T1 (*M* = 115.04, *SD* = 20.86) compared with *T2* (*M* = 101.74, SD = 20.80) and *T3* (M = 91.41, *SD* = 18.48; T2–T3 ns). The main effect of group and the interaction between group and time was not significant (*F*s < 2, *p*s > 0.1). For teacher-reported social responsiveness, the results of a repeated measures ANOVA showed no significant main effect of time or group (*F*s < 2. *ps* > 0.1). The interaction between time and group approached significance [F(2,33) = 2.97, *p* = 0.058, *η*_*p*_^2^ = 0.08], indicating that in the intervention group social responsiveness was marginally more improved at T3 compared with T1 (see Table 1).

#### Attentional Control

In order to check the validity of the flanker task, T1 response times (RTs) for correct responses for each trial type (congruent, neutral and incongruent) were explored using a repeated measures ANOVA. This analysis showed that there was a significant effect of condition [*F*(1.36, 46.12) = 74.23, *p* = < 0.01, *η*_*p*_^2^ = 0.67]. Post hoc analyses showed RTs for conflict trials were significantly slower (*M* = 951.53 ms), compared with both congruent (*M* = 751.26 ms) and neutral trials (*M* = 751.44; congruent and neutral trials ns). Analysis of errors in this task similarly showed a significant effect of condition [*F*(68, 1.65) = 34.62, *p* = < 0.01, *η*_*p*_^2^ = 0.50], highlighting more errors for the conflict trials (*M* = 3.2), compared with both congruent (*M* = 1) and neutral trials (*M* = 0.86; neutral congruent trials ns).

**Fig. 3:**
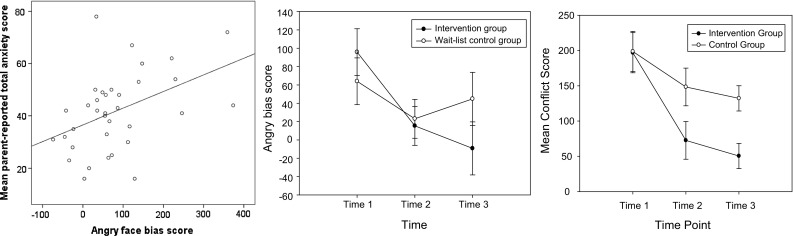
Correlation between parent-reported anxiety and angry face bias scores in the emotional face task (*left hand side*), angry face bias scores (*middle graph*) and conflict scores in the flanker task at for the intervention and wait-list control group at all three time points

Conflict scores on the flanker tasks (with higher scores indicative of greater interference) were analysed using a repeated measures ANCOVA (co-varying for IQ scores). This analysis showed a significant main effect of group [*F*(1, 31) = 6.92, *p* = 0.013, *η*_*p*_^2^ = 0.18], indicating that the overall mean conflict score for the intervention group (*M* = 106.65) was significantly lower than the control group (*M* = 159.74). There was no significant main effect of time and no interaction between time and group (*F* < 2, *p* > 0.1; see Fig. 3).

#### Attention to Threat

In order to understand baseline task performance in the schematic stroop task, T1 response times for upright and inverted faces were explored using a repeated measures ANOVA for 4 face (angry, fear, happy and neutral) by 2 orientation (upright and inverted). This analysis showed that there was no main effect of emotion or orientation (*F* < 1.5, *p* > 0.05). There was a significant interaction between emotion and orientation [*F*(3, 34) = 4.72, *p* < 0.01. *η*_*p*_^2^ = 0.12], highlighting that RTs to respond to upright angry faces (*M* = 955.60 ms) were greater, compared with fear (*M* = 916.62 ms), happy (*M* = 893.047 ms) and neutral faces (*M* = 902.66 ms; see Fig. 3); comparisons between all other faces were not significant. For the inverted control face stimuli there were no significant differences between RTs across condition (respective means for angry, fear, happy and neutral conditions were *M* = 902.63 ms, *M* = 923.34 ms, *M* = 903.02 ms and *M* = 887.73 ms respectively). The number of colour matching errors did not significantly differ for upright or inverted faces.

Bias scores on the schematic face stroop task (with positive scores indicating greater interference) were analysed using repeated measures ANOVA for 2 group (intervention and control) by 3 Face (angry, happy, fearful) and 3 Time (pre, post and follow-up). This analysis revealed a significant main effect of emotion [*F*(2, 32) = 9.37, *p* < 0.001, *η*_*p*_^2^ = 0.23]. Post hoc analyses using pairwise comparisons showed that bias scores for the angry trials were significantly higher (*M* = 38.97 ms), compared with fear (*M* = 9.43 ms) and happy trials (*M* = −7.26 ms), indicating that across the sample, participants experienced significantly greater interference from the angry faces (see Fig. 3).

## Discussion

Previous research has supported the efficacy of CBT interventions for use with an ASD population through clinic-based study and clinical samples (Lang et al. 2010). The aim of the current study was to extend this evidence base to investigate if CBT is an effective intervention to reduce symptoms of anxiety and social worry in a community derived sample of adolescents with ASD when delivered within a school setting. The use of a community sample allowed us to highlight that some adolescents attending mainstream school experience anxious affect (as reported via parents or teachers), and that identification of elevated anxiety symptoms had either gone unnoticed or untreated. Following a CBT intervention adolescents with ASD (versus a wait-list control group) showed greater reductions in anxiety symptoms, school anxiety and social worry, as reported by parents, teachers and young people themselves, and these results were maintained at a 6 week follow-up. In addition, teachers reported marginally increased social responsiveness for young people in the intervention group, which was most evident 6 weeks post-intervention. The impact of the intervention on attentional control and attention to threat was less clear; considering attention bias to threat, both groups showed reduced interference of threat stimuli to achieve task goals. In addition, the intervention group showed less distractibility overall, and although this difference was most marked post intervention and at follow up; this group difference was not sensitive to time.

The results of the current study support previous findings to show that CBT is an effective intervention for reducing symptoms of anxiety and social worry in a community sample of adolescents diagnosed with ASD, and where positive effects were maintained 6 weeks following the intervention. These improvements support the use of the Exploring Feelings intervention (Attwood 2000; 2004) in school settings and are consistent with the findings of previous studies in young people with ASD (e.g., McNally et al. 2013; Reaven et al. 2012; review by Kreslins et al. 2015). The intervention used in the current study was designed specifically for use with young people with ASD and the current results support recent modifications in the development of CBT interventions for this population, in terms of delivery and content (review by Moree and Davis 2010). Specifically, the intervention uses a coping model rather than a curative model (Beebe and Risi 2003) and a more directive teaching approach (Anderson and Morris 2006).

Previous findings suggest that for school-based interventions to be effective in terms of generalization and maintenance of effects, there is a need for teachers to incorporate strategies that promote these qualities (e.g., teaching new skills in natural settings and using everyday consequences to reinforce new behaviours; Machalicek et al. 2008). The generalization of target skills may require additional training for the teaching staff and parents. In the current study, front-line school staff were directly involved in the intervention delivery and followed the guidance given by the intervention facilitators to encourage adolescents to use taught skills across the school day. In addition, the intervention included homework tasks that also served to engage parents in the reinforcement of new skills. Reviews of CBT indicate that parent involvement in the delivery of interventions is as effective as individual or group based interventions (James et al. 2013). The inclusion of parents and teachers in the current study is reflected in the positive results across different sources; from adolescent self-report, as well as parents and teachers (see Chalfant et al. 2007, for similar findings). It suggests that skills taught within the CBT sessions resulted in symptom change that was evident across different contexts.

Consistent with previous research, the findings also showed that the intervention had a small positive effect on social skills more broadly, as reported by teachers (Storch et al. 2013; Sukhodolsky et al. 2008). Although the social responsiveness scale has previously been used to identify characteristics of ASD (rather than as an outcome measure), there is emerging evidence that it is a measure that is sensitive to change following treatment for individuals with ASD (Lopata et al. 2010; White et al. 2013). Previous research has, however, shown that young people with ASD can show difficulty in generalising taught social skills to contexts outside of the teaching environment (Bellini et al. 2007; White et al. 2007). These findings fit with the current study, which found that parents did not report change in social responsiveness following the intervention. There is a need for future research to systematically explore the impact of practice opportunities within the intervention environment and across different contexts to understand their importance in effecting and maintaining positive change for young people with ASD.

Previous research has shown that anxiety in children and adolescents is associated with poor attentional control and an attention bias to threat (review by Dudeney et al. 2015) and that this selective attention to threat is reduced following a CBT intervention (i.e., reduced attention to threat stimuli in the pursuit of task goals; Hadwin and Richards 2016; Reinholdt-Dunne et al. 2015). The current study also found that (parent-reported) anxiety was associated with increased interference of threat stimuli in an emotional stroop task and it supports the proposition that anxiety related threat biases are a feature of anxiety in adolescents with ASD. The current study however did not find links the CBT intervention with improvements in attentional control or a reduced bias to threat. The results showed that both groups showed increased resistance to distraction post-intervention and at follow-up; this was greater in the intervention group but this difference was not significantly associated with time. This result, if replicated with a larger sample size to demonstrate group differences over time, would fit well with a growing literature that has highlighted a bidirectional relationship between attentional processing and the regulation of anxious affect (e.g., Bishop 2007). In addition, it supports the proposition that re-appraisal can facilitate the regulation of negative affect, allowing individuals to meet task goals (Ochsner et al. 2002).

### Limitations and Future Directions

The current study represents a novel exploration to highlight the effectiveness of CBT in a community sample of adolescents in a school setting. It extends previous research to deliver the intervention in a context that was more familiar to adolescents and that showed beneficial effects on generalisation across contexts and functioning. The findings have implications for professionals working with children and adolescents with ASD with elevated anxiety to provide some guidance in contexts where interventions can be implemented to effect positive change. There are however several limitations linked to the current study. Firstly, the researchers did not formally measure treatment integrity. In addition, there was no active control group to explore whether CBT was more effective than other interventions and to control for time spent in the intervention. Moreover, researchers and raters were not blinded to condition allocation at post-intervention or follow-up, leading to potential over-reporting of change and active control groups can help to meet this difficulty. Finally, longer-term follow-up assessment of outcomes would yield useful information towards determining the durability of findings. Future research should consider exploring the efficacy and feasibility of a school-based intervention that aims to both reduce anxiety and increase social competency and attentional control. Moreover, larger sample sizes will allow greater consider of what mechanisms are important in understanding change.

## Footnotes

1 Please note that because of the exploratory nature of secondary outcomes, we have not corrected for multiple comparisons in this table.

2 Further analyses controlling for T1 anxiety scores in both analyses support this pattern of results, showing a main effect of group and indicating fewer anxiety symptoms for the intervention compared with the control group (all other effects were not significant).

3 Analyses controlling for T1 group differences in school anxiety scores showed a significant main effect of group, highlighting more anxiety symptoms at T1 and T3 in the control group compared with the intervention group.

## References

[CR1] American Psychiatric Association. (1994). *Diagnostic and statistical manual of mental disorders* (4th ed.). Washington, DC.

[CR2] American Psychiatric Association. (2013). *Diagnostic and statistical manual of mental disorders* (5th ed.). Washington, DC.

[CR3] Anderson S, Morris J (2006). Cognitive behaviour therapy for people with Asperger syndrome. Behavioural and Cognitive Psychotherapy.

[CR4] Attwood T (2000). Strategies for improving the social integration of children with Asperger syndrome. Autism.

[CR5] Attwood T (2004). Exploring feelings (anxiety).

[CR102] Beck AT (1993). Cognitive therapy: Past, present, and future. Journal of Consulting and Clinical Psychology.

[CR8] Beck AT, Rush AJ, Shaw BF, Emery G (1979). Cognitive therapy of depression.

[CR9] Beebe DW, Risi S, Reinecke MA, Dattilo FM (2003). Treatment of adolescents and young adults with high- functioning autism or Asperger syndrome. Cognitive therapy with children and adolescents: A casebook for clinical practice.

[CR10] Bellini S (2004). Social skill deficits and anxiety in high-functioning adolescents with autism spectrum disorders. Focus on Autism and Other Developmental Disabilities.

[CR12] Bellini S, Peters JK, Benner L, Hopf A (2007). A meta-analysis of school-based social skills interventions for children with autism spectrum disorders. Remedial and Special Education.

[CR13] Berument SK, Rutter M, Lord C, Pickles A, Bailey A (1999). Autism screening questionnaire: Diagnostic validity. The British Journal of Psychiatry.

[CR14] Bishop SJ (2007). Neurocognitive mechanisms of anxiety: An integrative account. Trends in Cognitive Neuroscience.

[CR15] Browning J, Osborne LA, Reed P (2009). Research section: A qualitative comparison of perceived stress and coping in adolescents with and without autistic spectrum disorders as they approach leaving school. British Journal of Special Education.

[CR16] Chalfant AM, Rapee R, Carroll L (2007). Treating anxiety disorders in children with high functioning autism spectrum disorders: A controlled trial. Journal of Autism and Developmental Disorders.

[CR17] Chandler S, Charman T, Baird G, Simonoff E, Loucas T, Meldrum D, Pickles A (2007). Validation of the social communication questionnaire in a population cohort of children with autism spectrum disorders. Journal of the American Academy of Child and Adolescent Psychiatry.

[CR105] Constantino JN, Gruber CP (2002). The Social Responsiveness Scale.

[CR106] Constantino JN, Davis SA, Todd RD, Schindler MK, Gross MM, Brophy S (2003). Validation of a brief quantitative measure of autistic traits: Comparison of the social responsiveness scale with the autism diagnostic interview-revised. Journal of Autism and Developmental Disorders.

[CR19] Department for Education. (2001). *Inclusive schooling children with special educational needs*. London. DfE. Retrieved 10 January 2013 from https://www.education.gov.uk/publications/eOrderingDownload/DfES-0774-2001.pdf.

[CR108] Dudeney, J., Sharpe, L., & Hunt, C. (2015). Attentional bias towards threatening stimuli in children with anxiety: A meta-analysis. *Clinical Psychology Review*, *40*, 66–75. doi:10.1016/j.cpr.2015.05.007. 10.1016/j.cpr.2015.05.007 26071667

[CR107] Eriksen CW, Schultz DW (1979). Information processing in visual search: A continuous flow conception and experimental results. Perception & Psychophysics.

[CR104] Garland, T. (2005). Test description. *Journal of Occupational Psychology, Employment and Disability, 7*(2), 131–134. Retrieved from http://webarchive.nationalarchives.gov.uk/20130703133823/http://www.dwp.gov.uk/docs/no2-05-test-review.pdf.

[CR25] Gillott A, Furniss F, Walter A (2001). Anxiety in high-functioning children with autism. Autism.

[CR27] Hadwin JA, Donnelly N, Richards A, French CC, Patel U (2009). Childhood anxiety and attention to emotion faces in a modified Stroop task. British Journal of Developmental Psychology.

[CR28] Hadwin JA, Richards HJ (2016). Working memory training and CBT reduces anxiety symptoms and attentional biases to threat: A preliminary study. Frontiers in Psychology.

[CR29] Humphrey N, Lewis S (2008). Make me normal: The views and experiences of pupils on the autistic spectrum in mainstream secondary schools. Autism.

[CR30] James AC, James G, Cowdrey FA, Soler A, Choke A (2013). Cognitive behavioural therapy for anxiety disorders in children and adolescents (Review). Cochrane Database Systematic Reviews.

[CR31] Kasari C, Rotheram-Fuller E, Locke J, Gulsrud A (2012). Making the connection: Randomized controlled trial of social skills at school for children with autism spectrum disorders. Journal of Child Psychology and Psychiatry.

[CR32] Kerns CM, Kendall PC (2012). The presentation and classification of anxiety in autism spectrum disorder. Clinical Psychology: Science and Practice.

[CR33] Kreslins, A., Robertson, A. E., & Melville, C. (2015). The effectiveness of psychosocial interventions for anxiety in children and adolescents with autism spectrum disorder: A systematic review and meta-analysis. *Child and Adolescent Psychiatry and Mental Health, 9*(22). doi:10.1186/s13034-015-0054-7. 10.1186/s13034-015-0054-7 PMC4482189 26120361

[CR34] Lang R, Regester A, Lauderdale S, Ashbaugh K, Haring A (2010). Treatment of anxiety in autism spectrum disorders using cognitive behaviour therapy: A systematic review. Developmental Neuro-rehabilitation.

[CR101] Lopata C, Thomeer ML, Volker MA, Toomey JA, Nida RE, Lee GK (2010). RCT of a manualized social treatment for high-functioning autism spectrum disorders. Journal of Autism and Developmental Disorders.

[CR36] Lyneham HJ, Street AK, Abbott MJ, Rapee RM (2008). Psychometric properties of the school anxiety scale—Teacher reported (SAS-TR). Journal of Anxiety Disorders.

[CR100] Machalicek, W., O’Reilly, M. F., Beretvas, N., Sigafoos, J., Lancioni, G., Sorrells, A., et al. (2008). Review: A review of school-based instructional interventions for students with autism spectrum disorders. *Research in Autism Spectrum Disorders*, *2*(3), 395–416. doi:10.1016/j.rasd.2007.07.001.

[CR103] Malowsky J, Mogg K, Bradley BP, McClure-Tone E, Ernst M, Pine DS, Monk CS (2010). A preliminary investigation of neural correlates of treatmetn in adolescents with generalised anxiety disorder. Journal of Child and Adolescent Psychopharmacology.

[CR38] McNally RH, Lincoln AJ, Brown MZ, Chavira DA (2013). The Coping Cat program for children with anxiety and autism spectrum disorder: A pilot randomized controlled trial. Journal of Autism and Developmental Disorders.

[CR39] Moree BN, Davis III TE (2010). Cognitive-behavioral therapy for anxiety in children diagnosed with autism spectrum disorders: Modification trends. Research in Autism Spectrum Disorders.

[CR40] National Institute of Clinical Excellence. (2013). *Social anxiety disorder nice guideline 159*. Retrieved 25 March 2014 from http://www.nice.org.uk/nicemedia/live/14168/63868/63868.pdf.

[CR41] Neil AL, Christensen H (2009). Efficacy and effectiveness of school-based prevention and early intervention programs for anxiety. Clinical Psychology Review.

[CR42] Ochsner KN, Bunge SA, Gross JJ, Gabrieli JDE (2002). Rethinking feelings: An fMRI study of the cognitive regulation of emotion. Journal of Cognitive Neuroscience.

[CR44] Reaven J, Blakeley-Smith A, Culhane-Shelburne K (2012). Group cognitive behavior therapy for children with high-functioning autism spectrum disorders and anxiety: A randomized trial. Journal of Child Psychology and Psychiatry.

[CR45] Reaven JA, Blakeley-Smith A, Nichols S, Dasari M, Flanigan E, Hepburn S (2009). Cognitive-behavioral group treatment for anxiety symptoms in children with high-functioning autism spectrum disorders: A pilot study. Focus on Autism and Other Developmental Disabilities.

[CR46] Reinholdt-Dunne L, Mogg K, Vangkilde SA, Bradley BP, Esbjørn BH (2015). Attention control and attention to emotional stimuli in anxious children before and after cognitive behavioral therapy. Cognitive Therapy and Research.

[CR47] Richards HJ, Benson V, Donnelly N, Hadwin JA (2014). Exploring the function of selective attention and hypervigilance for threat in anxiety. Clinical Psychology Review.

[CR48] Rotheram-Fuller E, MacMullen L (2011). Cognitive-behavioral therapy for children with autism spectrum disorders. Psychology in the Schools.

[CR50] Rueda MR, Posner MI, Rothbart MK, Davis-Stober CP (2004). Development of the time course for processing conflict: An event-related potentials study with 4 year olds and adults. BMC Neuroscience.

[CR51] Russell E, Sofronoff K (2005). Anxiety and social worries in children with Asperger syndrome. Australian and New Zealand Journal of Psychiatry.

[CR52] Rutter M, Bailey A, Lord C (2003). Social Communication Questionnaire.

[CR53] Rutter M, Le Couteur A, Lord C (2003). Autism Diagnostic Interview—Revised.

[CR54] Simonoff E, Pickles A, Charman T, Chandler S, Loucas T, Baird G (2008). Psychiatric disorders in children with autism spectrum disorders: Prevalence, comorbidity, and associated factors in a population-derived sample. Journal of the American Academy of Child and Adolescent Psychiatry.

[CR55] Sofronoff K, Attwood T, Hinton S (2005). A randomised controlled trial of a CBT intervention for anxiety in children with Asperger syndrome. Journal of Child Psychology and Psychiatry.

[CR56] Spence S (1995). Social skills training: Enhancing social competence with children and adults.

[CR57] Spence SH (1998). A measure of anxiety symptoms among children. Behaviour Research and Therapy.

[CR58] Storch EA, Arnold EB, Lewin AB, Nadeau JM, Jones AM, De Nadai AS (2013). The effect of cognitive-behavioral therapy versus treatment as usual for anxiety in children with autism spectrum disorders: A randomized, controlled trial. Journal of the American Academy of Child and Adolescent Psychiatry.

[CR59] Sukhodolsky DG, Bloch MH, Panza KE, Reichow B (2013). Cognitive-behavioral therapy for anxiety in children with high-functioning autism: A Meta-analysis. Pediatrics.

[CR60] Sukhodolsky DG, Scahill L, Gadow KD, Arnold LE, Aman MG, McDougle CJ (2008). Parent-rated anxiety symptoms in children with pervasive developmental disorders: Frequency and association with core autism symptoms and cognitive functioning. Journal of Abnormal Child Psychology.

[CR61] Sze KM, Wood JJ (2007). Cognitive behavioral treatment of comorbid anxiety disorders and social difficulties in children with high-functioning autism: A case reported. Journal of Contemporary Psychotherapy.

[CR63] Waddington, E. M., & Reed, P. (2006). Parents’ and local education authority officers’ perceptions of the factors affecting the success of inclusion of pupils with autistic spectrum disorders. *International Journal of Special Education*, *21*(3), 151–164. Retrieved from http://files.eric.ed.gov/fulltext/EJ843627.pdf.

[CR64] Waters AM, Wharton TA, Zimmer-Gembeck MJ, Craske MG (2008). Threat-based cognitive biases in anxious children: Comparison with non-anxious children before and after cognitive behavioural treatment. Behaviour Research and Therapy.

[CR65] Wechsler D (1999). Wechsler abbreviated scale of intelligence.

[CR66] White SW, Keonig K, Scahill L (2007). Social skills development in children with autism spectrum disorders: A review of the intervention research. Journal of Autism and Developmental Disorders.

[CR67] White SW, Ollendick T, Albano AM, Oswald D, Johnson C, Southam-Gerow MA (2013). Randomized controlled trial: Multimodal anxiety and social skill intervention for adolescents with autism spectrum disorder. Journal of Autism and Developmental Disorders.

